# Identification of a hotspot on PD-L1 for pH-dependent binding by monoclonal antibodies for tumor therapy

**DOI:** 10.1038/s41392-020-00254-z

**Published:** 2020-08-24

**Authors:** Hongchuan Liu, Xiaoshan Bi, Yuehua Zhou, Rui Shi, Sheng Yao, Jianxun Qi, Hui Feng, Meiqing Feng, Jinghua Yan, Shuguang Tan

**Affiliations:** 1 Department of Biological Medicines & Shanghai Engineering Research Center of Immunotherapeutics, School of Pharmacy, Fudan University, 201203 Shanghai, China; 2 Department of Antibody Discovery and Engineering, Shanghai Junshi Biosciences Co., Ltd, 201203 Shanghai, China; 3 School of Life Sciences, Anhui University, 230601 Hefei, China; 4 CAS Key Laboratory of Microbial Physiological and Metabolic Engineering, Institute of Microbiology, Chinese Academy of Sciences, 100101 Beijing, China; 5 CAS Key Laboratory of Pathogenic Microbiology and Immunology, Institute of Microbiology, Chinese Academy of Sciences, 100101 Beijing, China

**Keywords:** Structural biology, Immunology

**Dear Editor,**

Monoclonal antibody (mAb)-based tumor immune checkpoint therapy (ICT) has gained particular interest in recent years.^1^ The molecular basis of binding between mAbs and PD-1 or PD-L1 has been reported, providing clear information of the binding “hotspots” for mAbs.^2,3^ Tumor suppression efficacy of PD-L1 specific mAbs relies on not only the blocking of PD-1/PD-L1 interaction to restore T cell reactivity, but also Fc-mediated tumor cell cytotoxity. PD-L1 antibody drug conjugate (ADC) for selective chemo-guided immune modulation of tumor has also been developed which has shown promising tumor suppression potency.^4^ MAbs that could bind to antigen in a pH-dependent manner would improve recycling of the antibodies and engineered IL-6R mAbs with pH-dependent binding properties have displayed increased lysosomal delivery and therapeutic potency.^5^ However, no PD-L1 specific mAb with pH-dependent binding property has been reported, and whether the binding to a specific region on PD-L1 would induce pH-dependent interaction remains unknown.

Here we report the binding properties of a PD-L1 specific antibody JS003 with tumor suppression potency. JS003 is a humanized PD-L1 specific mAb which could block the binding of PD-L1 to PD-1 or CD80 and showed a binding affinity (*K*_D_) of 2.88 × 10^−10^ M in surface plasmon resonance (SPR) analysis (Fig. 1a, b; supplementary Fig. S1a). The ability of JS003 to promote T cell reactivity in vitro was investigated with mixed leukocyte reactions (MLR) assay. The results revealed that JS003 has substantially enhanced the allogeneic T cell response as measured by IL-2 and IFN-γ secretion (Fig. 1c and supplementary Fig. 1b). The in vivo tumor suppression efficacy of JS003 was examined in human PD-L1 knock-in mice of the C57BL/6 background (C57/hPD-L1) with syngeneic MC38-hPD-L1 tumor cells. The results showed that inhibition of tumor growth was observed in a dose dependent manner with significant anti-tumor efficacy in 3 mg/kg and 10 mg/kg JS003 treatment groups compared with PBS group at the end of the observation period (Day 27) (*p* < 0.001), while the low dose group (1 mg/kg) showed no significant change in tumor size compared to negative control PBS group (*p* = 0.07) (Fig. 1d and supplementary Fig. 2).

**Fig. 1:**
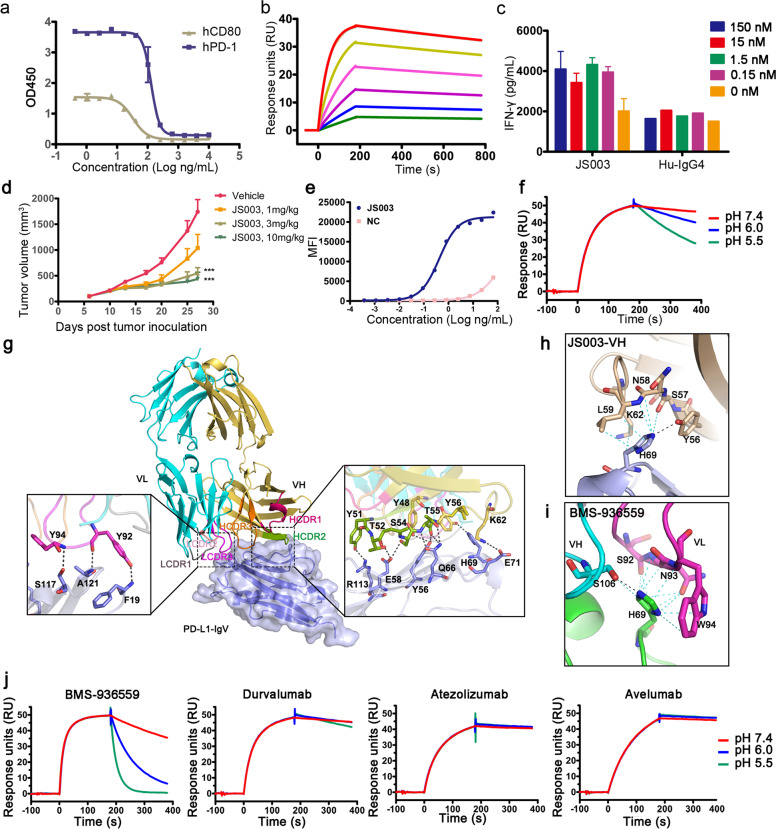
Functional characteristics of JS003 and molecular basis for pH binding dependency of PD-L1 specific mAbs. **a** ELISA based assay to test the blocking efficacy of JS003 to the interaction of PD-L1 with PD-1 or CD80 as indicated. **b** SPR analysis of the binding profiles of JS003 to PD-L1. JS003 was immobilized on the chip and a serial dilutions of PD-L1 from 0.75nM to 24nM were then flowed through. **c** Enhanced cytokine production of T cells stimulated with allogeneic human dendritic cells in the presence of varied concentrations of JS003 as indicated. The concentration of IFN-γ was measured with ELISA assay. A humanized IgG4 was enrolled as negative control. **d** The in vivo tumor suppression efficacy of JS003 in hPD-L1 knock-in mice of the C57BL/6 background by inoculation of MC38-hPD-L1 tumor cell line. JS003 was injected i.p. every 3 or 4 days from day 6 with four doses, 1mg/kg, 3mg/kg, and 10mg/kg after MC38 tumor inoculation. Saline was enrolled as negative control. The data with each dot show the average tumor volume of the group while the SE was presented as longitudinal bars. **e** PD-L1 internalization upon binding to JS003 was measured with a pH-sensitive cyanine dye derivative CypHer5E platform with hPD-L1-expressing CHO-K1 cells. A serial dilutions of JS003 and negative control (Hu-IgG4) mAbs were labeled with CypHer5E and incubated with hPD-L1-expressing CHO-K1 cells. The detected fluorescence indicates the internalization of PD-L1 into the acidic endosomal vesicles inside the cells. **f** The binding kinetics of JS003 to PD-L1 under different pH conditions was monitored by using SPR. JS003 antibodies were captured on biosensors and associated with recombinant human PD-L1 in a buffer of pH 7.4. The dissociation was then followed either at pH 7.4, 6.0 or 5.5. **g** The complex structure of JS003 and PD-L1. The CDR1, CDR2, and CDR3 loops of the heavy chain (HCDR1, HCDR2, and HCDR3) and light chain (LCDR1, LCDR2, and LCDR3) are colored differently as indicated. The right panel showed the detailed binding of VH fragment to PD-L1, while the left panel showed the detailed binding of VL fragment to the PD-L1. Residues involved in the hydrogen bond interaction are shown as sticks and labeled. Hydrogen bonds are shown as dashed black lines. **h**–**i** The amino acids involved in the interaction between H69 of PD-1 and JS003 (**h**) or BMS-936559 (**i**). **j** The binding kinetics of BMS-936559, durvalumab, atezolizumab, and avelumab to PD-L1 under different pH conditions was monitored by using SPR as in **f**. The results presented here were representative of three independent experiments with similar results.

We next examined the ability of the PD-L1-specific JS003 to induce PD-L1 internalization using a pH-sensitive cyanine dye derivative CypHer5E in hPD-L1-expressing CHO-K1 cells. The results showed that JS003 has induced PD-L1 internalization with an EC_50_ of 0.42 nM (Fig. 1e). The binding kinetics of JS003 under different pH conditions were further investigated by using SPR. JS003 antibodies were captured on a biosensor and the association with PD-L1 proteins was monitored in a neutral buffer of pH 7.4, while the dissociation was followed at pH 7.4, 6.0 or 5.5. JS003 exhibited a dissociation rate 3.4-fold and 8.7-fold faster at pH 6.0 and pH 5.5 than that at pH 7.4, respectively (Fig. 1f and supplementary Table 1). These results indicate that JS003 has high affinity to PD-L1 at the neutral pH but may rapidly dissociates with PD-L1 at lower pH in the endosome. Internalized JS003 could be degraded in the acidic endosome. The rapid dissociation in lower pH would enable antigen dissociation in endosome and reduce the possibility of degradation in lysosome, and facilitate recycling of the antibody for reuse.

To elucidate the binding mechanisms of JS003 to PD-L1, the complex structure of JS003-Fab/PD-L1 was determined at a resolution of 2.0 Å (Fig. 1g, supplementary Table 2). The structure analysis revealed that JS003 utilizes both of its heavy (H) and light (L) chain to interact with PD-L1. Of note, the binding of JS003 H chain is mainly attributed to HCDR2 that residues from HCDR2 (Y48, Y51, T52, S54, T55, and Y56) and frame region (K62) of JS003 H chain have formed hydrogen bond interactions with amino acids from C and C′ strands (Y56, E58, Q66, H69, and E71) and F strand (R113) of PD-L1 (Fig. 1g and supplementary Table 3). Additionally, Y92 and Y94 from LCDR3 have formed hydrogen bond interactions with I2, S117 and A121 of PD-L1. Superimposition of the structure of JS003/PD-L1 complex with PD-1/PD-L1 complex (PDB code: 4ZQK) revealed that the binding of JS003 to PD-L1 shows a stereo clash with that of PD-1 and the binding surface of JS003 and PD-1 is substantially overlapped (supplementary Fig. 3).

The pH-dependent binding properties of JS003 is of particular interest. Previous studies revealed that pH-dependent binding of the mAbs is usually mediated by the presence of histidine in the CDRs that interacts with the antigen. However, there is no histidine in the CDRs of JS003 that contacts PD-L1. Therefore, we further analyzed the residues in PD-L1 that interact with JS003. The analysis revealed that the only histidine involved in the interaction between JS003 and PD-L1 is H69, which has formed multiple van der Waals interactions with amino acids from HCDR2 (Y56, S57, N58, L59 and K62) (Fig. 1h and supplementary Table 3). Moreover, H69 also formed a hydrogen bond interaction with Y56 of HCDR2 (Fig. 1h). Therefore, we speculate that the interaction with H69 in PD-L1 is responsible for the pH-dependent binding properties of JS003.

To further validate this hypothesis, we analyzed whether H69 is also involved in the interaction with other mAbs with complex structures available. The binding orientations of these mAbs and their binding areas on PD-L1 varied (supplementary Fig. 4). The complex structure of BMS-936559/PD-L1 showed that H69 of PD-L1 has formed not only multiple van der Waals interactions with amino acids from HCDR3 (S106) and LCDR3 (S92, N93, and W94), but also hydrogen bond interaction with S106 of HCDR3 (Fig. 1i). On the other hand, no substantial interactions with H69 of PD-L1 were observed for other PD-L1 specific mAbs, i.e., avelumab, durvalumab, and atezolizumab (supplementary Fig. 5). Therefore, we speculate that the binding of BMS-936559 to PD-L1 may also be affected by pH conditions. We then assessed the pH binding dependency of BMS-936559, which forms multiple interactions with H69 of PD-L1, together with durvalumab, atezolizumab, and avelumab, which do not bind to H69 of PD-L1, with methods described above. As is expected, the results revealed that the dissociation rate of BMS-936559 in pH 5.5 (*K*_d_ = 1.67 × 10^−3^ s^−1^) is 25.5-fold faster than that in pH 7.4 (*K*_d_ = 4.25 × 10^−2^ s^−1^) (Fig. 1j and supplementary Table 1). On the other hand, the dissociation rate at varied pH conditions showed no substantial differences with durvalumab, avelumab or atezolizumab. Therefore, the pH binding dependency to H69 of PD-L1 may be a common property for PD-L1 specific mAbs. The engineered tocilizumab rapidly dissociates IL-6R at pH 6.0, which enables pH-dependent antigen dissociation in the acidic endosomes and reduces IL-6R-mediated clearance of the antibodies.^5^ Therefore, pH-dependent binding property of the mAbs could offer significant advantages over conventional antibodies and enable better pharmacokinetics in clinical treatment of the tumors. However, the clinical benefits of PD-L1 mAbs with pH-dependent binding properties still need further evaluations.

In summary, JS003 is a PD-L1 specific mAb with substantial tumor suppression efficacy. Rapid dissociation of JS003 with PD-L1 was observed in acidic conditions and structural analysis revealed that H69 of PD-L1 may have played a critical role. The pH-dependent binding property by targeting H69 of PD-L1 was further confirmed with BMS-936559. H69 may serve as a hotspot for future design of PD-L1 specific biologics with pH-dependent binding properties.

## Supplementary information

Supplementary Material
