# Supplementary material for: Identification of a hotspot on PD-L1 for pH-dependent binding by monoclonal antibodies for tumor therapy
Source: Signal Transduct Target Ther. 2020 Aug 24;5:158. doi: 10.1038/s41392-020-00254-z (PMC7445246; doi:10.1038/s41392-020-00254-z)
Supplement: Supplementary file 1 — Supplementary Material [file 41392_2020_254_MOESM1_ESM.docx]

**Supplemental Material for**

**Identification of a hotspot on PD-L1 for pH-dependent binding by monoclonal antibodies for tumor therapy**

Hongchuan Liu^1,5,#^, Xiaoshan Bi^2,3, #^, Yuehua Zhou^5^, Rui Shi^3^, Sheng Yao^5^, Jianxun Qi^4^, Hui Feng^5^, Meiqing Feng^1,*^, Jinghua Yan^2,3,4,*^, Shuguang Tan^2,4,*^

Correspondence e-mail: fmq@fudan.edu.cn; [yanjh@im.ac.cn](mailto:yanjh@im.ac.cn); tansg@im.ac.cn.

**Supplementary information, Figures and Tables**

Fig.S1 Specific binding of JS003 with PD-L1 and enhanced IL-2 production upon stimulation with JS003.

Fig.S2 Individual follow-up of tumor size for JS003 treatment.

Fig.S3 Competitive binding of JS003 and PD-L1 with PD-1.

Fig.S4 Comparison of the binding of anti-PD-L1 mAbs.

Fig.S5 Location of the H69 of PD-L1 in the complex structures of PD-1/PD-L1 or mAbs/PD-L1.

Table S1. pH-dependent antigen binding properties of JS003, BMS-936559, durvaluab, avelumab and atezolizumab.

Table S2. Crystallographic data collection and refinement statistics..

Table S3. Residues contributed interaction between JS003 and PD-L1

**Supplementary information, Materials and Methods**

**Supplementary information, ReferencesSupplementary Information**

**
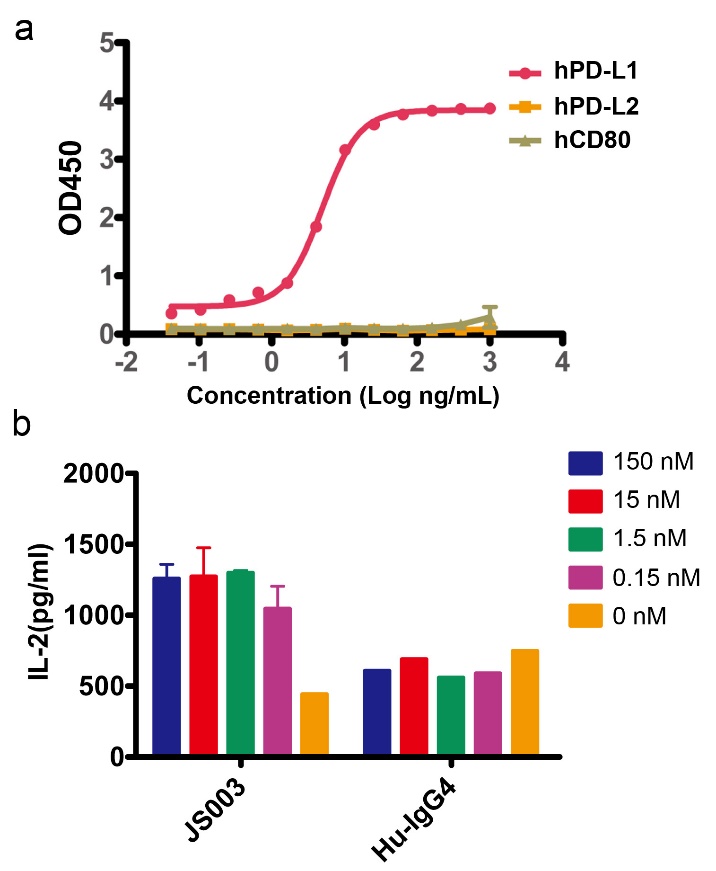
**

**Supplementary Fig. 1 Specific binding of JS003 with PD-L1 and enhanced IL-2 production upon stimulation with JS003. (a)** ELISA-based binding of JS003 to PD-L1. A serial concentrations of JS003 was added to ELISA wells pre-coated with PD-L1. Wells pre-coated with PD-L2 and CD80 were detected in parallel as negative control. **(b)** Enhanced cytokine production of T cells stimulated with allogeneic human DC in the presence of varied concentrations of JS003 as indicated. The concentration of IL-2 were measured with ELISA assay. A humanized IgG4 was enrolled as negative control.


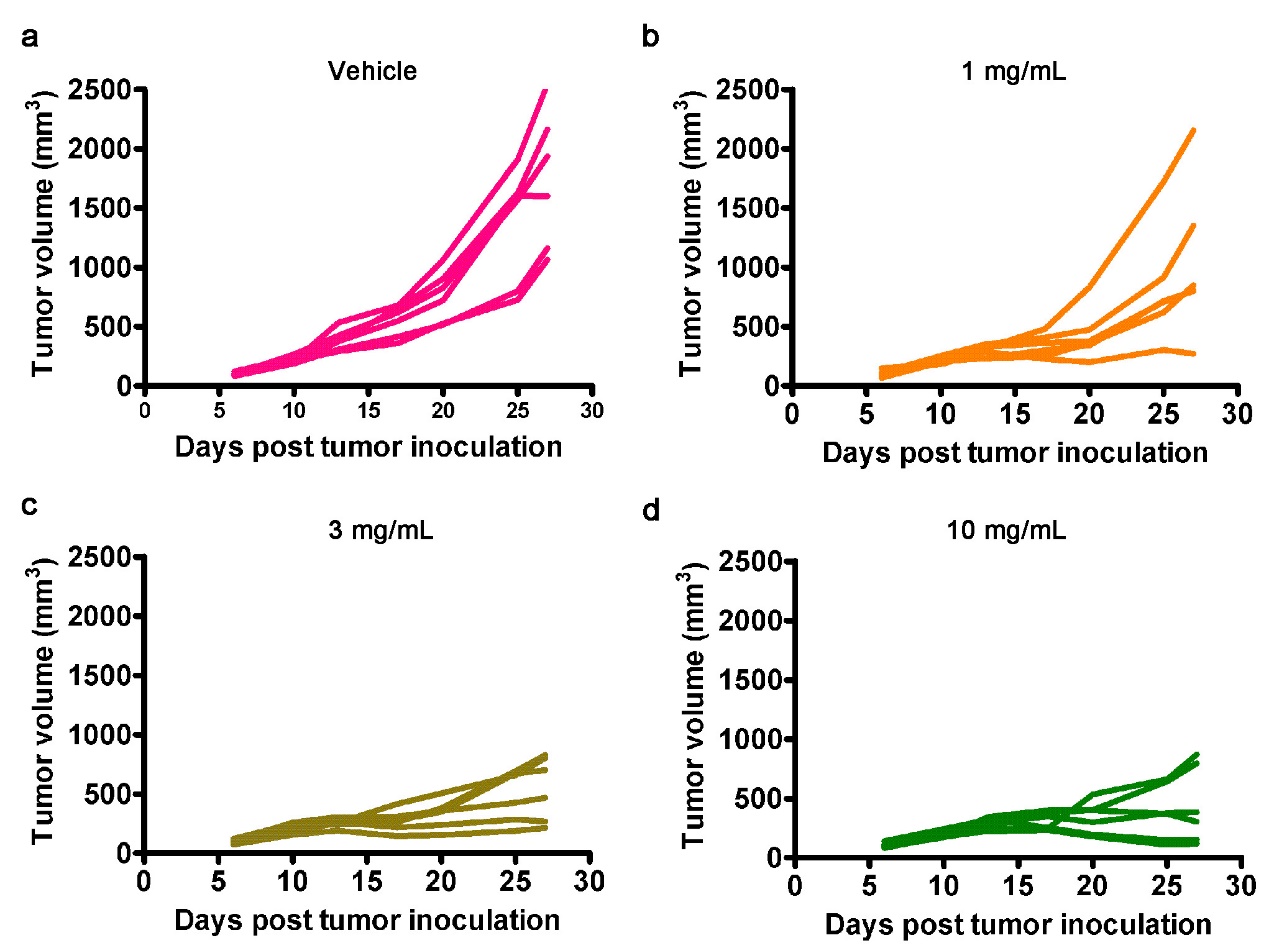


**Supplementary Fig. 2** **Individual follow-up of tumor size for JS003 treatment.** Individual follow-up of tumor sizes is presented for each experimental group with each line showing the changes of the tumor size of each mouse. There are six mice in each treatment group.

**
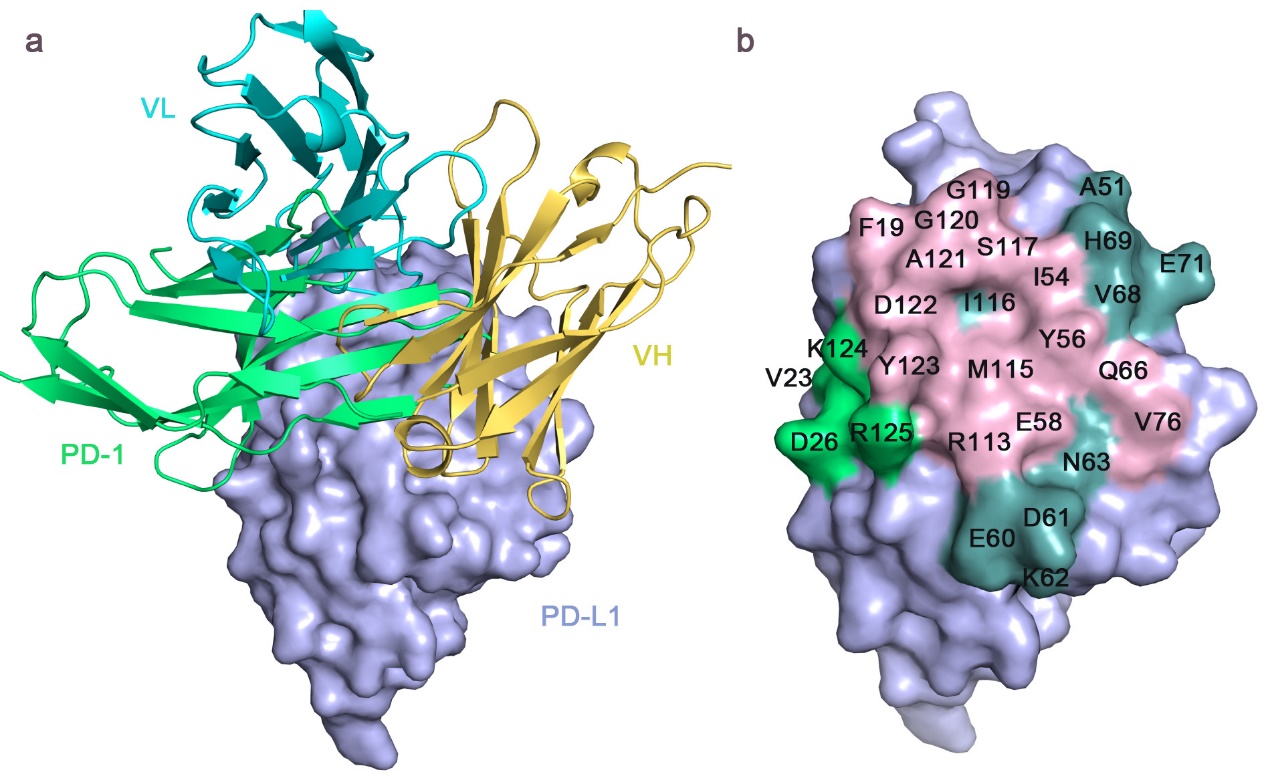
 Supplementary Fig. 3 Competitive binding of JS003 and PD-L1 with PD-1. (a)**. Superposition of the JS003/PD-L1 complex structure with PD-1/PD-L1 complex structure (PDB code: 4ZQK). PD-1 is shown in lemon-green while VH of JS003 is colored in orange and VL in cyan. **(b).** Binding surface of PD-L1 with PD-1 or JS003. The residues in contact with PD-1 are colored in lemon green, whereas residues in contact with JS003 are colored in deep teal, and the overlapping residues bound by both PD-1 and JS003 are colored in light pink.


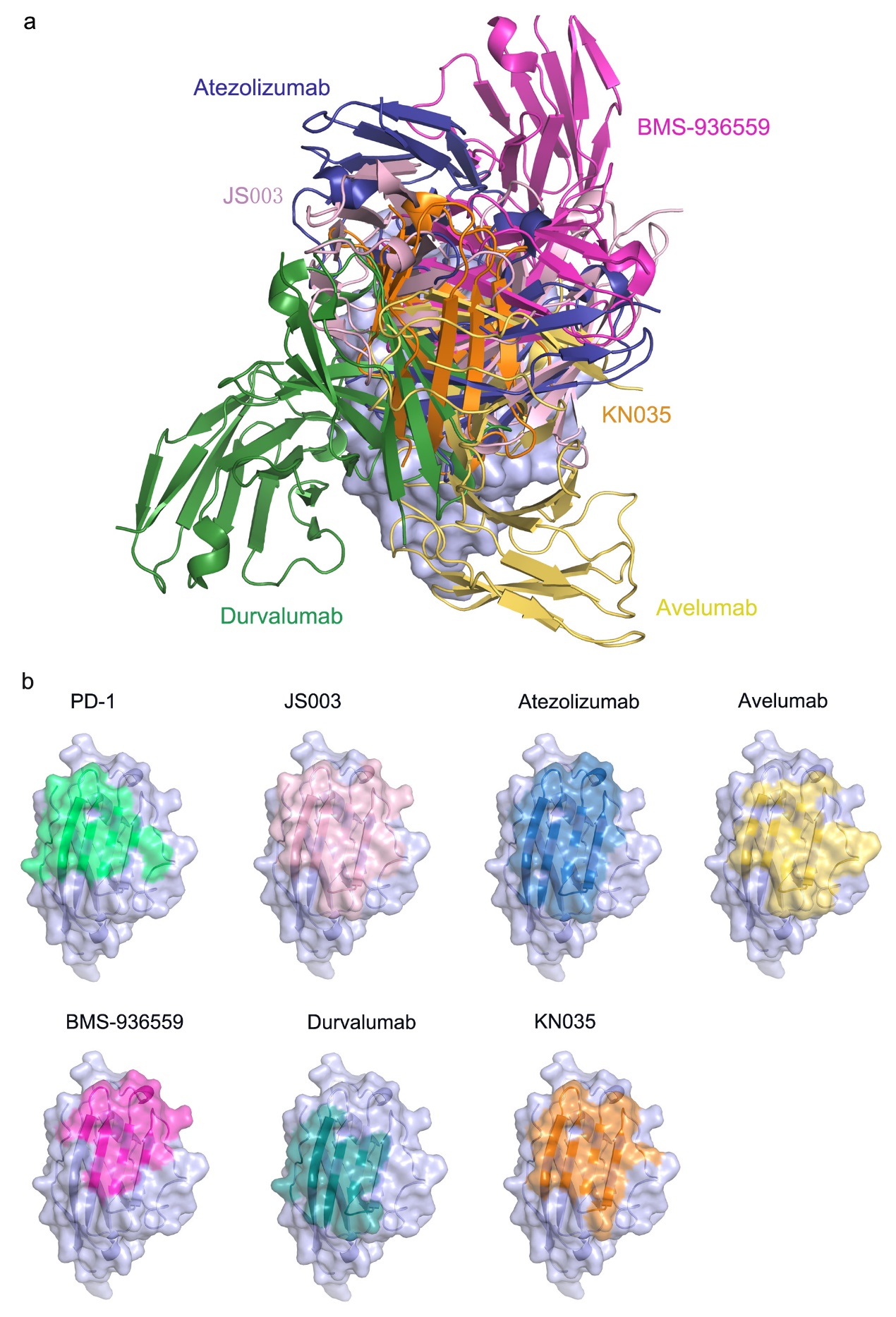


**Supplementary Fig. 4 Comparison of the binding of anti-PD-L1 mAbs. A.** Superimposition of the structures of PD-L1 complexed with the indicated mAbs. PD-L1 is shown as a surface diagram in light blue, while the mAbs are shown as cartoon in different colors as indicated. **B.** Binding surface of PD-1 and binding epitopes of JS003, atezolizumab, avelumab, BMS-936559, durvalumab and nanobody KN035 on PD-L1.


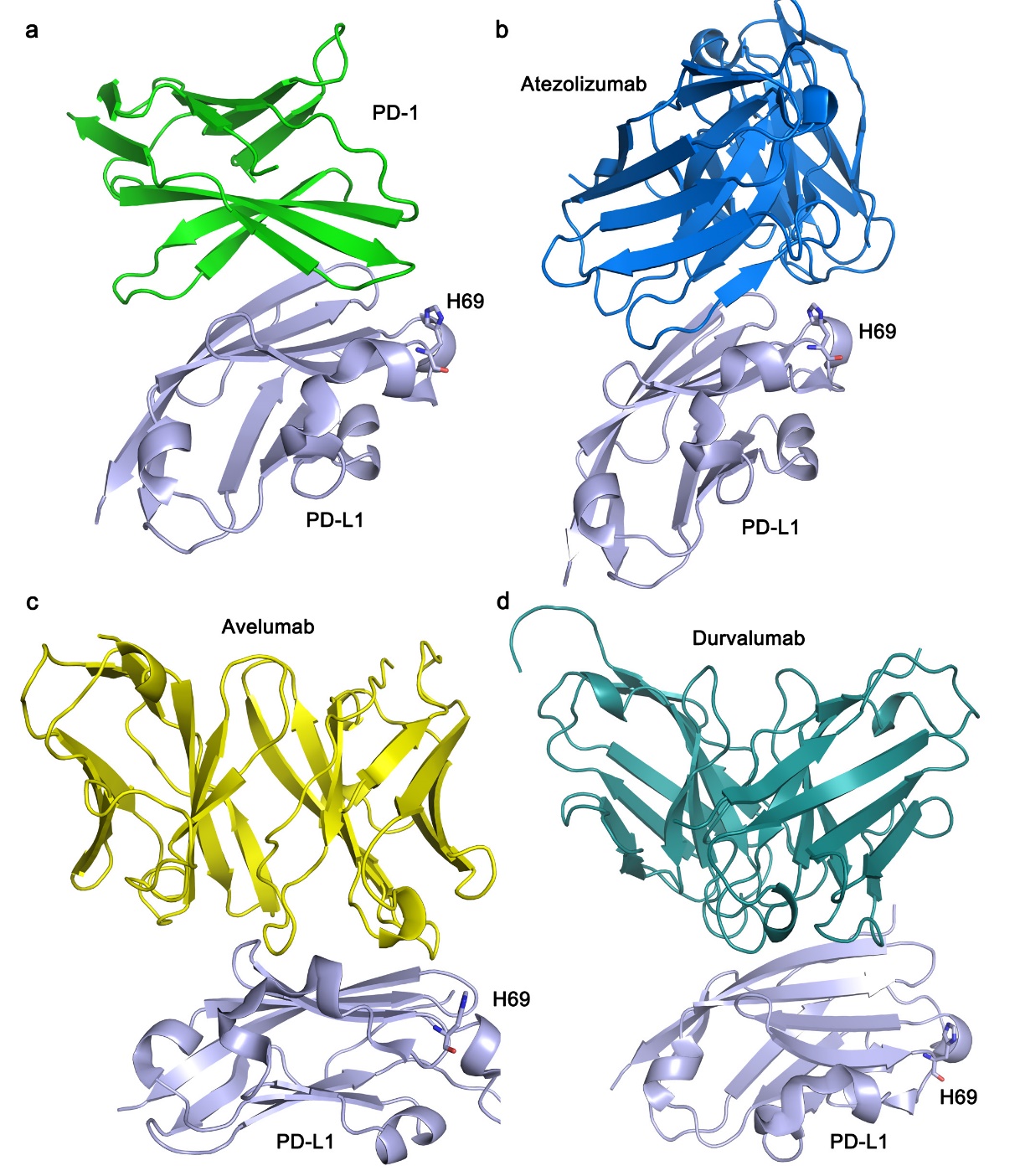


**Supplementary Fig. 5 Location of the H69 of PD-L1 in the complex structures of PD-1/PD-L1 or mAbs/PD-L1.** The complex structures of PD-1/PD-L1 (A), atezolizumab/PD-L1 (B), Avelumab/PD-L1 (C) and Durvalumab/PD-L1 (D) are presented as cartoon with H69 in PD-L1 shown in sticks and labeled accordingly.

**Supplementary Table 1. pH-dependent antigen binding properties of JS003, BMS-936559, durvaluab, avelumab and atezolizumab**

| Antibody | pH 7.4 | pH 6.0 | | pH 5.5 | |
| --- | --- | --- | --- | --- | --- |
|  | *k*_d_ (s^−1^) | *k*_d_ (s^−1^) | *k*_d_ ratio  pH 6.0/ pH 7.4 | *k*_d_ (s^−1^) | *k*_d_ ratio  pH 5.5/ pH 7.4 |
| JS003 | 3.57 × 10^-4^ | 1.23 × 10^-3^ | 3.4 | 3.10 × 10^-3^ | 8.7 |
| BMS-936559 | 1.67 × 10^-3^ | 1.08 × 10^-2^ | 6.5 | 4.26 × 10^-2^ | 25.5 |
| durvalumab | 2.83 × 10^-4^ | 4.71 × 10^-4^ | 1.7 | 8.91 × 10^-4^ | 3.1 |
| avelumab | 1.23 × 10^-4^ | 1.35 × 10^-4^ | 1.1 | 1.94 × 10^-4^ | 1.6 |
| atezolizumab | 1.60 × 10^-4^ | 1.93 × 10^-4^ | 1.2 | 2.64 × 10^-4^ | 1.7 |

**Supplementary Table 2. Crystallographic data collection and refinement statistics.**

|  | JS003/PD-L1 |
| --- | --- |
| **Data collection** |  |
| Space group | P 1 21 1 |
| Wavelength (Å) | 0.97890 |
| Unit cell dimensions |  |
| a, b, c (Å) | 107.98, 65.64, 107.53 |
| α, β, γ(°) | 90.00, 114.47, 90.00 |
| Resolution (Å) | 41.58-2.00 |
| No. reflections | 86422 |
| *R_merge_* | 0.133/1.023 |
| *I/σ* | 1.74(2.00) |
| Completeness (%) | 92.6 |
| Redundancy | 5.3(5.5) |
| **Refinement** |  |
| Resolution (Å) | 41.58-2.00 |
| *R_work_ / R_free_* | 0.222/0.258 |
| No. atoms | 8992 |
| Protein | 8334 |
| Ligands | 0 |
| Water | 658 |
| **R.m.s. deviations** |  |
| Bond lengths (Å) | 0.0008 |
| Bond angles (°) | 0.678 |
| **Ramachandran plot** |  |
| Favored (%) | 98.78 |
| Allowed (%) | 1.22 |
| Outliers (%) | 0.00 |

*Values in parentheses are for highest-resolution shell.

**Supplementary Table 3. Residues contributed interaction between JS003 and PD-L1**

| **JS003** | **PD-L1** | **Contacts** | **Total** |
| --- | --- | --- | --- |
| **H chain** |  |  | 211 |
| Y31 | M115 | 1**^1^** |  |
| Y48 | Y56 | 7 (1)^2^ |  |
| S50 | E58 | 1 |  |
| Y51 | E60, D61, R113 | 4, 5, 8 (1) |  |
| T52 | E58, E60, D61, K62, N63, R113 | 9 (1), 1, 10, 5, 6, 1 |  |
| G53 | V76 | 4 |  |
| S54 | Y56, E58, N63, Q66, V76 | 33, 3(1), 4, 2 |  |
| T55 | Y56, Q66, V68 | 5(1), 17(3), 1 |  |
| Y56 | I54, Y56, V68, H69 | 5, 5, 2, 10(1) |  |
| S57 | V68, H69 | 1, 2 |  |
| N58 | H69 | 2 |  |
| L59 | A51, H69 | 1, 14 |  |
| K62 | V68, H69, E71 | 7, 2, 5(1) |  |
| W99 | Y56, M115, I116, S117, A121, D122, Y123 | 6, 19, 7, 4, 2, 4, 3 |  |
| L100 | A121, D122, Y123 | 5, 3, 2 |  |
| **L chain** |  |  | 41 |
| I2 | G 119 | 1 |  |
| Y92 | F19, G120, A121 | 5(1), 7, 7(1) |  |
| G93 | S117, G119, G120, A121 | 2, 2, 2, 2 |  |
| Y94 | I54, S117, G119 | 2, 9(1), 2 |  |

^1^ Numbers represent the number of atom-to-atom contacts between JS003 and PD-L1 residues, which were analyzed by the Contact program in CCP4 suite (the distance cutoff is 4.5Å).

^2^ Numbers in the parentheses represent the number of hydrogen bonds between JS003 and PD-L1 residues which were analyzed by the Contact program in CCP4 suite (the distance cutoff is 3.5 Å).

**Material and methods**

**Plasmid construction and protein purification**

The DNA sequence encoding the extracellular domain of human PD-L1 with six histidines at the C-terminus was cloned into CAGa vector (in-house constructed) with *NheI* and *XhoI* restriction sites. Human PD-L1-His-tag was expressed in 293-Freestyle cells (ATCC) and purified by His-Trap HP column (GE Healthcare). The DNA sequence encoding the extracellular domain of human PD-L1with Fc region of human IgG1 at the C-terminus, extracellular domains of human PD-1 and CD80 with Fc region of mouse IgG2a, were cloned individually into HX1 vector (in-house constructed) with *SapI* restriction site. PD-L1-hFc, PD-1-mFc, CD80-mFc proteins were expressed in human 293-Freestyle cells and purified by HiTrap Protein A HP column(GE Healthcare).

The coding sequences for heavy chain and light chain of durvaluab, avelumab, atezolizumab and BMS-936559 were obtained from IMGT database (INN:10010), IMGT database (INN:10062), IMGT database (INN:9814) and PDB database (ID:5GGT), respectively. The H chain of durvaluab, avelumab, atezolizumab and BMS-936559 were cloned individually into HX1 vector (in-house constructed) with SapI restriction site，and the L chain were cloned individually into HX2 vector (in-house constructed) with SapI restriction site. Durvaluab, avelumab, atezolizumab and BMS-936559 proteins were expressed in human 293-Freestyle cells and purified by HiTrap Protein A HP column (GE Healthcare).

Gene encoding the ectodomain of human PD-L1 was subcloned into pET-21a vector and transformed into *E.coli* BL21(DE3) as inclusion bodies. *E.coli* cells grown in LB medium at 37 ^o^C, 200 rpm, and was induced with 0.5 mM IPTG once the density reached 0.4-0.6 at 600 nm. After further culture for 5 hours, it was collected by centrifugation and disrupted by high pressure cracker. The inclusion bodies was extracted from cell lysate and was dissolved in dissolution buffer (6 M Gua-HCl ,10％Glycerin 50 mM Tris pH8.0 ,100 mM NaCl, 10mM EDTA, 10 mM DTT ) to 30 mg/mL. The dissolved inclusion bodies was added drop-wise into refolding buffer containing 100 mM Tris pH8.0, 400 mM L-Arg HCl, 2 mM EDTA, 5 mM GSH and 0.5 mM GSSG for 20 hours at 4°C as previously described ^1^. The refolded protein was exchanged into the buffer of 20 mM Tris pH8.0, 50 mM NaCl, and purified by HiLoad^TM^16/600 Superdex^TM^200pg (GE Healthcare). Full-length JS003 proteins were obtained from Shanghai Junshi Biosciences. The purified mAbs were digested with the Human IgG Fab and F(ab’)_2_ Preparation Kits (Thermo Scientific) according to the manufacturer’s instructions. The protein fragments were purified by HiTrap Protein A FF (GE Healthcare), and then exchanged to the buffer of 20 mM Tris pH 8.0, 150 mM NaCl.

**SPR binding characterization of JS003 to human PD-L1**

Binding characteristics of JS003 to PD-L1 was performed on Biacore T200 system with series S sensor chip CM5 (GE Healthcare, Catalog No. BR100530) at 25˚C. For surface preparation, goat anti-human IgG Fc antibodies (Jackson Immuno Research) was immbolilzed on both flow cells of CM5 sensor chip, with the first flow cell as the reference channel, the second flow cell as the test channel. Serially diluted recombinant human PD-L1-His-tag proteins (0.75 nM, 1.5 nM, 3 nM, 6 nM, 12 nM and 24 nM, with 24 nM in duplicate) were then injected through both channels. For each cycle, after antigen association and dissociation, the sensor surface was regenerated with 10 mM glycine-HCl (pH 1.5). Kinetic binding data was analyzed with Biacore T200 Evaluation Software (Version 3.0) using 1:1 binding model.

**ELISA-based binding of JS003 to human PD-L1, PD-L2 and CD80**

ELISA-based binding assay was performed to evaluate the specific binding of JS003. Recombinant human PD-L1 (with six histidines at the C-terminus), PD-L2 (with hFc region at the C-terminus, Sino Biology) or human CD80-mFc, was immobilized to microtiter plates for 90 min, washed, and blocked using 2% BSA for 90min. Varied concentrations of JS003 (a 2.5-fold dilution series from 1 μg/mL to 0.1 ng/mL and a blank control) were allowed to bind for one hour and then washed with PBST. Bound JS003 was detected by incubating with mouse anti-human IgG4 Fc-HRP secondary antibody (Southern Biotech). The concentration that gave half-maximal effect (EC_50_) was determined using a log (agonist) vs. response-variable slope curve fit (GraphPad Prism).

**Blocking assay of PD-L1 and its receptor PD-1 or CD80**

ELISA-based and flow cytometry-based blocking assays were performed. Biotin labeled human PD-L1-hFc fusion protein were added at 0.3 μg/mL to the plate immobilized with streptavidin (Jackson Immuno Research) for 40 min and then washed. PD-1-mFc and CD80-mFc fusion proteins were diluted to 3 μg/mL and incubated with JS003 antibody with a 2.5-fold dilution series from 10 μg/mL to 1.05 ng/mL and a blank control. Then the mixture was added to the wells with PD-L1 proteins and the bound PD-1-mFc or CD80-mFc fusion protein were detected with anti-mouse IgG (Fc Specific)-peroxidase antibody (Sigma, Catalog NO. A2554). Half-maximal inhibitory concentrations (IC_50_) of JS003 were determined using a log(inhibitor) vs. response – variable slope curve fit (GraphPad Prism).

**Stimulation of T cell reactivity with mixed leukocyte reaction assay**

Mixed leukocyte reaction (MLR) was performed to evaluate T cell reactivation activity of JS003. CD4^+^ T cells were isolated from PBMCs (AllCells) using EasySep human CD4^+^ T cells enrichment kit (StemCell Technologies). Dendritic cells (DCs) were generated by incubating PBMCs (AllCells) first with IL-4 (1000 U/ml) and GM-CSF (1000 U/ml), followed by maturation in media containing tumor necrosis factor (1000 U/ml), IL-1β (5 ng/ml), IL-6 (10 ng/ml), and prostaglandin E2 (1 μM) for 2 days. 1×10^4^ DCs and 1×10^5^ CD4 ^+^ T cells were seeded in RPMI 1640 medium (Gibco) in a 96-well plate and incubated with serial 10-fold dilutions of JS003 or control Hu-IgG4 antibody from a starting concentration of 150 nM overnight. The concentration of IL-2 in culture supernatants was measured by a Cisbio kit (Cisbio) 3 days later with two duplicated wells for each sample. The concentration of IFN-γin culture supernatants was measured by a Cisbio kit (Cisbio) 5 days later with two duplicated wells for each sample.

***In vivo* anti-tumor activity in a syngeneic tumor model**

The animal experiments were performed in accordance with regulations for care and use of laboratory animals at Immune Technology Corp., and were approved by Immune Technology’s Institutional Animal Care and Use Committee.

For this syngeneic model, the genetically modified mouse strain with human PD-L1 knocked-in was employed. Human PD-L1 knock-in mice were purchased from Jiangsu Biocytogen Co., Ltd (strain: C57BL/6-*PdCd274^tm1(hCD274)^*/Bcgen). All mice were kept in specific pathogen-free conditions, mice were subcutaneously inoculated with 1×10^6^ MC38-hPD-L1 cells in 100 uL phosphate-buffered saline on day 0. On day 6, the inoculated mice were randomized into six groups (tumor volume averages 20–70 mm^3^) and treated with JS003 at 1, 3, and 10 mg/kg via intraperitoneal injection twice a week. The tumor growth was monitored twice a week and the volume of the tumors was calculated by the formula: ½ length × width^2^.

**Internalization of PD-L1 assay**

JS003-induced PD-L1 internalization was evaluated using a pH-sensitive cyanine dye derivative CypHer5E (GE Healthcare, Catalog NO. PA15401) which is minimally fluorescent at basic/neutral pH and maximally fluorescent at an acidic pH and is, therefore, ideally suited to report the movement of a receptor from the cell surface into internal acidic endosomes. CypHer5E-labeled JS003 binds PD-L1 on the surface of cells, but no fluorescence is detected until the antibody/PD-L1 complex is internalized into the acidic endosomal vesicles inside the cells. JS003 and anti-hu-IgG4 antibodies were labeled with CypHer5E according to manufacturer’s instructions. The labeled antibodies were separated from unconjugated dye by size-exclusion centrifugal filter and stored at -30°C until use. CHO PD-L1 cells were incubated in culture media (Ham’s F-12 supplemented with 10% FBS) for 4 hours with varying concentrations (0.4pM-66.7nM, 3-fold dilution) of CypHer5E-labeled antibodies at 37°C. Cells were then washed with cold media and analyzed by flow cytometry (APC channel). Data analysis was performed with FlowJo and GraphPad Prism.

**pH-dependent antigen binding assay**

pH-dependent antigen binding analysis of JS003, durvaluab, avelumab, atezolizumab and BMS-936559 were performed by using Biacore T200 system with series S sensor chip CM5 (GE Healthcare, Catalog No. BR100530) at 25˚C. For surface preparation, goat anti-human IgG Fc antibodies (Jackson Immuno Research) was immbolilzed on both flow cells of CM5 sensor chip, with the first flow cell as the reference channel, the second flow cell as the test channel. For the capture phase, 2 μg/mL of JS003, 1 ug/mL of durvaluab or BMS-936559, was individually captured on CM5 sensor chip to around 150 RU in buffer (pH 7.4). For the association phase, 24 nM of PD-L1 (with six histidines at the C-terminus) interacted with the antibody captured on the CM5 sensor chip and reached about 49 RU in buffer (pH 7.4). As for atezolizumab, 1 μg/mL of antibody was captured to around 143 RU, 24 nM of PD-L1 was then injected to about 42 RU. As for avelumab, 1 μg/mL of antibody was captured to around 143 RU, 36 nM of PD-L1 was then injected to about 46 RU. Dissociation was monitored in solutions of different pH (7.4, 6.0 and 5.5). The pH 7.4 buffer contains 10 mM HEPES (pH 7.4), 150 mM NaCl, 3 mM EDTA and 0.05% P20. The pH 6.0 buffer contains 10 mM MES (pH 6.0), 150 mM NaCl, 3 mM EDTA and 0.05% P20. The pH 5.5 buffer contains 10 mM NaAc (pH 5.5), 150 mM NaCl and 0.05% P20. Data analysis of dissociation rate (*k*_d_ (s^−1^)) was determined using Biologic Scrubber version 2.

**Crystallization, data collection and structure determination**

The *E.coli* cell-expressed PD-L1 protein and JS003-Fab were mixed at molar ratio of 1:1 and incubated on ice for 1 hour, the complex was subsequently purified using GE. PD-L1/JS003-Fab was concentrated to 10mg/ml and used for crystal screening by sitting-drop vapor diffusion method at 4 where 0.8 μl of the protein solution was mixed with 0.8 μl of reservoir solution. Diffraction-quality crystals of PD-L1-IgV/JS003-Fab were obtained in a buffer with 0.1 M Na HEPES 7.0, 15% w/v PEG 20000 at 18 ˚C.

Crystals were cryoprotected in 20% glycerol in the mother liquor and flash-cooled in liquid nitrogen. The diffraction data were collected at Shanghai Synchrotron Radiation Facility (SSRF) BL19U, and all data were processed with HKL2000^2, 3^. The complex structure was solved by molecular replacement method using phase with the reported PD-L1 structure and avelumab structure (PDB: 5GRJ) as the search models ^3^. Subsequent model building and refinement were performed using Coot and Phenix to refine the results, respectively ^5, 6^. The stereochemical qualities of the final model were assessed with MolProbity. Data collection and refinement statistics are summarized in Table S1. All structural figures were generated using Pymol (<http://www.pymol.org>).

**Data deposition**

Atomic coordinates have been deposited in the Protein Data Bank (PDB, http://www.rcsb.org/pdb) under accession code 7C88.

**References**

1 Chen Y, Liu P, Gao F, Cheng H, Qi J, Gao GF. A dimeric structure of PD-L1: functional units or evolutionary relics? *Protein Cell* 2010; **1**:153-160.

2 Otwinowski Z, Minor W. Processing of X-ray diffraction data collected in oscillation mode. *Method Enzymol* 1997; **276**:307-326.

3 Read RJ. Pushing the boundaries of molecular replacement with maximum likelihood. *Acta Crystallogr D* 2001; **57**:1373-1382.

4 Bailey S. The Ccp4 Suite - Programs for Protein Crystallography. *Acta Crystallographica Section D* 1994; **50**:760-763.

5 Emsley P, Cowtan K. Coot: model-building tools for molecular graphics. *Acta Crystallogr D Biol Crystallogr* 2004; **60**:2126-2132.

6 Adams PD, Afonine PV, Bunkóczi G *et al.* PHENIX: a comprehensive Python-based system for macromolecular structure solution. *Acta Crystallographica Section D* 2010; **66**:213-221.
